# Family Impact, Caregiver Burden, Parental Monitoring, and Glycemic Control in Children with Type 1 Diabetes: A Cross-Sectional Study

**DOI:** 10.3390/children13091253

**Published:** 2026-09-15

**Authors:** Bahadir Yazicioglu, Tugba Kontbay-Cetin

**Affiliations:** 1 Department of Family Medicine, Samsun City Hospital, Samsun 55090, Türkiye; 2 Department of Pediatric Endocrinology, Samsun City Hospital, Samsun 55090, Türkiye; tugbakontbay@gmail.com

**Keywords:** type 1 diabetes, caregiver burden, family impact, parental monitoring, glycemic control

## Abstract

**Highlights:**

**What are the main findings?**

- Greater parental monitoring was associated with lower HbA1c, including after adjustment for perceived household financial adequacy and in the age-restricted sensitivity analysis.
- Families reporting income below expenses had higher HbA1c levels and greater diabetes-specific family impact and caregiver burden than financially balanced families.

**What are the implications of the main findings?**

- Parental monitoring and household socioeconomic circumstances should both be considered when evaluating glycemic and family-related outcomes in pediatric type 1 diabetes.
- Family-centered assessment may help identify families experiencing greater diabetes-related impact and caregiver burden, particularly those reporting financial strain.

**Abstract:**

**Objectives:** To examine the association between family-related psychosocial factors and glycemic control in children with type 1 diabetes. **Methods:** This cross-sectional study included 67 children with type 1 diabetes and their parents. Data were collected using the Diabetes Family Impact Scale, Caregiver Burden Scale, and Parental Monitoring of Diabetes Care Scale. Sociodemographic and clinical variables, including glycated hemoglobin (HbA1c) levels, were recorded. Spearman correlation analyses and multivariable regression models were performed. **Results:** A total of 67 parent–child dyads were included. Diabetes-specific family impact was strongly correlated with caregiver burden (ρ = 0.617, *p* < 0.001). Participants reporting household income below expenses had higher HbA1c levels (8.14 ± 1.85 vs. 7.07 ± 0.93, *p* = 0.002), higher DIFS scores (21.05 ± 8.55 vs. 14.29 ± 9.23, *p* = 0.005), and higher CBS scores (33.72 ± 14.35 vs. 26.13 ± 11.60, *p* = 0.022) than those reporting income equal to expenses. Higher parental monitoring was associated with lower HbA1c in the bivariate analysis (ρ = −0.533, *p* < 0.001) and remained associated after adjustment for perceived household financial adequacy (β = −0.580, *p* < 0.001). Perceived household financial adequacy was also associated with HbA1c in the adjusted model (β = −0.273, *p* = 0.002). **Conclusions:** Greater parental monitoring was consistently associated with lower HbA1c, while perceived household financial inadequacy was associated with poorer glycemic control and greater diabetes-specific family impact and caregiver burden. These findings highlight the relevance of both family and socioeconomic factors in pediatric type 1 diabetes care. Given the cross-sectional design, the observed associations should not be interpreted as causal.

## 1. Introduction

Type 1 Diabetes Mellitus (DM) is a chronic disease characterized by the autoimmune destruction of pancreatic β cells [1]. This phenomenon impacts millions of individuals globally [2]. According to the IDF Diabetes Atlas 2025, an estimated 9.15 million individuals worldwide were living with diagnosed clinical type 1 diabetes in 2024 [3]. In addition to its clinical burden, type 1 diabetes imposes substantial and ongoing demands on children and their caregivers, with important implications for family life and caregiver well-being [4]. Adequate management is imperative to circumvent deleterious long-term complications, including but not limited to cardiovascular disease, renal disease, ischemic stroke, and vision loss [2].

Insulin replacement therapy remains the cornerstone of treatment for type 1 diabetes [2]. Acute complications include diabetic ketoacidosis (DKA) and hypoglycemia, while long-term complications include microvascular and macrovascular disease. DKA is particularly important in children because it may be present at diagnosis and can be life-threatening [5]. In addition to these clinical complications, the ongoing management of type 1 diabetes may be accompanied by important psychosocial challenges that extend beyond glycemic control alone [6].

Children with type 1 diabetes, particularly at younger ages, depend substantially on their parents for daily diabetes management. Parents are responsible for multiple aspects of care, including insulin administration, glucose monitoring, nutrition, physical activity, and the prevention and management of hypoglycemia. These continuous demands may affect family life, increase financial and psychosocial strain, and interfere with parents’ occupational and social roles. Consequently, caring for a child with type 1 diabetes may impose a substantial caregiver burden encompassing physical, psychological, social, and financial dimensions [4,7,8].

Caregiver burden is a multidimensional concept that may vary according to the social and cultural context in which care is provided. It encompasses the emotional, physical, psychological, social, and financial difficulties experienced by family members providing care [9]. It also reflects caregivers’ responses to the demands and limitations of caregiving and the imbalance between care needs and their other responsibilities [10].

Diabetes management extends beyond pharmacological treatment and requires sustained engagement in daily self-management behaviors. Patient-centered approaches addressing behavioral and social factors have been associated with improved self-management outcomes in diabetes [11].

Recent studies from Türkiye have separately demonstrated associations of parental monitoring with glycemic control, caregiver burden with family and disease-related characteristics, and socioeconomic disadvantage with inequalities in pediatric diabetes management and access to diabetes technologies [12,13,14]. These findings indicate that the individual components examined in the present study are not novel in isolation. However, diabetes-specific family impact, caregiver burden, parental monitoring, perceived household financial adequacy, and glycemic control have rarely been evaluated simultaneously within the same clinical sample. The contribution of the present study is therefore integrative, providing a multidimensional assessment of how these family and socioeconomic factors coexist in pediatric type 1 diabetes. Accordingly, the primary aim was to examine the associations of diabetes-specific family impact, caregiver burden, and parental monitoring with glycemic control in children with type 1 diabetes. Secondary aims were to investigate the relationships of sociodemographic and clinical characteristics with family impact and caregiver burden. We hypothesized that greater parental monitoring would be associated with lower HbA1c levels and that greater socioeconomic disadvantage would be associated with higher family impact and caregiver burden.

## 2. Materials and Methods 

### 2.1. Research Design and Setting

The present study was conducted at the Pediatric Endocrinology Outpatient Clinic of Samsun Training and Research Hospital, employing a single-center, cross-sectional, analytical design. The hospital is a tertiary referral center serving both urban and rural populations in Samsun and the surrounding region of northern Türkiye. The data collection period spanned a three-month interval from January to March of 2025.

### 2.2. Study Population and Sample Flow

At the inception of the study, the number of pediatric patients with type 1 diabetes expected to visit the outpatient clinic during the predefined three-month study period was estimated to be 75. The actual number of eligible clinic visits during this period was 83. A total of 69 parents consented to participate in the study. Two participants were excluded because of incomplete questionnaire data. Consequently, the final analytical sample consisted of 67 parent–child dyads. A consecutive sampling approach was used, with eligible families presenting to the outpatient clinic during the study period invited to participate.

### 2.3. Inclusion and Exclusion Criteria

The inclusion criteria for this study are as follows: subjects must be between 6 and 18 years of age, have a diagnosis of Type 1 DM for at least 6 months, undergo regular follow-up at the center where the study was conducted, and have written informed consent from a parent/guardian.

No additional exclusion criteria were applied beyond failure to meet the inclusion criteria or incomplete questionnaire data.

### 2.4. Data Collection

Data were collected during outpatient clinic visits using parent-completed questionnaires and medical record review. Sociodemographic information and the study scales were completed by the participating parent/caregiver, while clinical data were obtained from the child’s medical records. All three scales were completed by the participating parent/caregiver; the children did not complete any of the study questionnaires.

Sociodemographic data included the child’s and parent’s age, sex, educational status, employment status, family structure, perceived household financial adequacy, and place of residence.

Clinical data: Clinical information, including duration of type 1 diabetes, insulin delivery modality, and the most recent HbA1c value, was obtained from the child’s medical records. Parental awareness of the child’s most recent HbA1c value was assessed separately by asking the participating parent whether they knew this value.

Diabetes Family Impact Scale (DIFS): The DIFS was developed by Katz et al. [15] to assess the diabetes-specific impact of childhood type 1 diabetes on the family. The scale consists of 14 items covering four domains: school, work, finances, and family well-being. Items are rated on a four-point Likert scale, with higher scores indicating a greater negative impact of diabetes on the family. The Turkish version was validated by Cetintas and Akgun-Kostak [16] in parents of children aged 6–18 years with type 1 diabetes and retained the original four-domain, 14-item structure. In the present study, the DIFS was completed by the participating parent/caregiver, and the total score was used as a continuous measure of diabetes-specific family impact.

Caregiver Burden Scale (CBS): Caregiver burden was assessed using the 22-item scale originally developed by Zarit et al. [17] to evaluate the burden experienced by individuals providing care. Each item is rated on a five-point Likert scale from 0 to 4, yielding a total score ranging from 0 to 88, with higher scores indicating greater perceived caregiver burden. The Turkish version was validated by Inci and Erdem [18]. In the present study, the scale was completed by the participating parent/caregiver and was used as a continuous measure of perceived caregiver burden associated with caring for a child with type 1 diabetes.

Parental Monitoring of Diabetes Care Scale (PMDCS): Parental monitoring of diabetes management was assessed using the 27-item scale developed and subsequently revised by Ellis et al. [19]. The instrument assesses parental supervision and monitoring of diabetes-care behaviors and is rated on a five-point Likert scale. Total scores range from 27 to 135, with higher scores indicating greater parental monitoring. The Turkish version was validated by Turk et al. [20] for use in adolescents with type 1 diabetes. In the present study, the scale was completed by the participating parent/caregiver and the total score was analyzed as a continuous measure of parental monitoring.

Because the Turkish version of the PMDCS was validated in parents of adolescents aged 12–18 years, its use in younger children was considered a potential measurement limitation. Therefore, an age-restricted sensitivity analysis was performed among participants aged 12–17 years, corresponding to the validated age range represented in our sample.

### 2.5. Statistical Analysis

Statistical analyses were performed using Jamovi version 2.6.22.0. Continuous variables were summarized as mean ± standard deviation (SD), and categorical variables as frequencies and percentages. Distributional assumptions were assessed using the Shapiro–Wilk test. Internal consistency of the DIFS, CBS, and PMDCS was evaluated using Cronbach’s alpha coefficients. Because several continuous variables were non-normally distributed and educational and financial adequacy variables were ordinal, bivariate associations were evaluated using Spearman’s rank correlation coefficient. Comparisons between the two represented perceived household financial adequacy groups (income below expenses and income equal to expenses) were performed using Welch’s independent-samples *t*-test, with Hedges’ g reported as the effect-size measure.

Multivariable linear regression analyses were performed to examine factors associated with HbA1c, DIFS, and CBS scores. Predictor variables were selected based on clinical and theoretical relevance. The DIFS and CBS models included PMDCS score, perceived household financial adequacy, child age, and diabetes duration. The HbA1c model included PMDCS score and perceived household financial adequacy as prespecified predictors. HbA1c was log-transformed for regression analysis because of its non-normal distribution and regression diagnostics. HC3 heteroscedasticity-consistent robust standard errors were used. Regression results are presented as unstandardized coefficients (B), robust standard errors (SE), standardized coefficients (β), 95% confidence intervals (CI), and *p* values. Overall model fit was evaluated using the F statistic and adjusted R^2^, and multicollinearity was assessed using variance inflation factors (VIFs).

Because the Turkish version of the PMDCS was originally validated in adolescents with type 1 diabetes, a sensitivity analysis was additionally performed among participants aged 12–17 years. In this age-restricted subgroup, the association between PMDCS and HbA1c was examined using Spearman’s rank correlation and the same multivariable HbA1c regression model including PMDCS score and perceived household financial adequacy.

Analyses addressing the prespecified study hypotheses were considered primary, whereas additional sociodemographic and subgroup analyses were considered secondary or exploratory. Given the number of statistical comparisons, findings from secondary and exploratory analyses were interpreted cautiously, with emphasis on effect estimates and 95% confidence intervals in addition to *p* values. All tests were two-sided, and *p* < 0.05 was considered statistically significant.

### 2.6. Ethical Approval

The study was approved by the Samsun Education and Research Hospital Non-Interventional Clinical Research Ethics Committee on 9 June 2021 (Decision No. GOKA/2021/11/3). The research was conducted in accordance with the principles of the Declaration of Helsinki. Written informed consent was obtained from all participating parents or legal guardians. Separate written or verbal assent was not obtained from the children or adolescents.

## 3. Results

The study’s sample population included 67 children and their respective families. The sample included 56.7% female children (n = 38), with an average age of 10.9 ± 2.1 years and an age range of 6–17 years. The majority of the questionnaires were completed by mothers (85.1%). The mean age of mothers was 37.5 ± 4.8 years, while the mean age of fathers was 41.3 ± 4.9 years. While the majority of mothers were not employed (88.1%), a significant proportion of fathers were employed (91.0%). A subsequent examination of educational attainment revealed that the predominant educational level among both mothers and fathers was high school. A predominant family structure was identified, with 85.1% of the families exhibiting a nuclear family configuration. The mean number of children per family was 2.39 ± 0.88 (min: 1, max: 5), and the majority of families had 2 children (47.0%, n = 31). In 64.2% of families, income was lower than expenditure, while in 35.8% income was equal to expenditure; no families reported income exceeding expenditure. With respect to the subject’s place of residence, the district center was identified as the predominant location (47.8%). The sociodemographic characteristics of the participants are presented in Table 1.

The duration of Type 1 DM diagnosis in patients was 6.55 ± 3.01 years. A significant proportion of the patient population, specifically 91.1%, utilized daily insulin injections as part of their therapeutic regimen. The mean HbA1c level was 7.76 ± 1.66. Furthermore, 67.2% of parents reported that they were unaware of their child’s most recent HbA1c value. The clinical characteristics of the participants are delineated in Table 2.

The scales utilized in the study were found to be satisfactory and demonstrated substantial internal consistency. The mean PMDCS score was 111.0 ± 13.0. The mean scores and reliability coefficients for the psychosocial scales are presented in Table 3.

Spearman’s rank correlation analysis was used to examine bivariate associations among sociodemographic, clinical, and psychosocial variables. DIFS was strongly positively correlated with CBS (ρ = 0.617, *p* < 0.001) and negatively correlated with father age (ρ = −0.377, *p* = 0.002) and perceived household financial adequacy (ρ = −0.330, *p* = 0.006). Greater perceived household financial adequacy was also associated with lower CBS scores (ρ = −0.243, *p* = 0.047) and lower HbA1c levels (ρ = −0.263, *p* = 0.032). Higher PMDCS scores were strongly associated with lower HbA1c levels (ρ = −0.533, *p* < 0.001). No significant correlations were observed between HbA1c and DIFS or CBS. The complete correlation matrix is presented in Table 4.

Perceived household financial adequacy was significantly associated with HbA1c, DIFS, and CBS scores. Participants from households reporting income below expenses had higher HbA1c levels (8.14 ± 1.85 vs. 7.07 ± 0.93, *p* = 0.002), higher DIFS scores (21.05 ± 8.55 vs. 14.29 ± 9.23, *p* = 0.005), and higher CBS scores (33.72 ± 14.35 vs. 26.13 ± 11.60, *p* = 0.022) than those reporting income equal to expenses. These group comparisons are summarized in Table 5.

Multivariable regression analyses were performed to examine factors associated with glycemic control, diabetes-specific family impact, and caregiver burden (Table 6). In the model with log-transformed HbA1c as the dependent variable (n = 67), higher PMDCS scores were associated with lower HbA1c (B = −0.0089, β = −0.580, *p* < 0.001), and greater perceived household financial adequacy was also associated with lower HbA1c (B = −0.112, β = −0.273, *p* = 0.002). The overall model was statistically significant and explained 41.2% of the adjusted variance in log-transformed HbA1c (adjusted R^2^ = 0.412, F (2,64) = 24.12, *p* < 0.001).

In the age-restricted sensitivity analysis including participants aged 12–17 years (n = 30), higher PMDCS scores remained significantly associated with lower HbA1c levels in the bivariate analysis (Spearman ρ = −0.519, *p* = 0.003). This association also remained significant in the multivariable model adjusted for perceived household financial adequacy (standardized β = −0.534, *p* < 0.001). Perceived household financial adequacy was also independently associated with HbA1c in this subgroup (standardized β = −0.330, *p* = 0.018). The overall model was statistically significant (F (2, 27) = 12.40, *p* < 0.001; adjusted R^2^ = 0.440).

In the DIFS model, financially balanced household status was independently associated with lower DIFS scores (B = −7.848, β = −0.407, *p* = 0.001), whereas PMDCS score, child age, and diabetes duration were not significantly associated with DIFS. The overall DIFS model was statistically significant but explained a modest proportion of the variance (adjusted R^2^ = 0.107, F (4,62) = 2.971, *p* = 0.026). The overall CBS model was not statistically significant (adjusted R^2^ = 0.018, F (4,62) = 1.296, *p* = 0.281). Although perceived household financial adequacy showed a nominally significant coefficient within this model (B = −7.834, β = −0.274, *p* = 0.040), this finding was considered exploratory given the nonsignificant overall model.

## 4. Discussion

The present study examined the relationships between diabetes-specific family impact, caregiver burden, parental monitoring, perceived household financial adequacy, and glycemic control in families of children with type 1 diabetes. Three main findings emerged. First, families reporting income below expenses had higher HbA1c levels and greater diabetes-specific family impact and caregiver burden than financially balanced families. Second, diabetes-specific family impact was strongly correlated with caregiver burden. Third, higher parental monitoring was associated with lower HbA1c in both bivariate and adjusted analyses, and this association remained significant in the age-restricted sensitivity analysis. In addition, financially balanced household status remained associated with lower DIFS scores in the multivariable model, whereas the overall CBS model was not statistically significant. These findings indicate that socioeconomic circumstances and family-related factors are associated with both glycemic control and the family experience of pediatric type 1 diabetes.

Type 1 diabetes imposes a substantial psychosocial burden on affected children and their families [21]. Family-related burden and disruptions in family functioning have also been reported among adolescents with type 2 diabetes [22]. The onset and ongoing management of childhood diabetes require substantial parental involvement and may be accompanied by considerable practical, emotional, and psychosocial challenges for parents and families [23,24]. In the present study, DIFS and CBS scores were strongly positively correlated (ρ = 0.617, *p* < 0.001), indicating that greater diabetes-specific impact on family functioning was associated with greater perceived caregiver burden. Because of the cross-sectional design, however, this relationship should be interpreted as an association rather than evidence of a directional or causal effect.

Parental involvement is an important component of diabetes management; however, the relationship between parental monitoring and glycemic control should be interpreted cautiously. In the present study, higher PMDCS scores were associated with lower HbA1c levels in the bivariate analysis (Spearman ρ = −0.533, *p* < 0.001). This association remained statistically significant after adjustment for perceived household financial adequacy (standardized β = −0.580, *p* < 0.001). Importantly, the association was also observed in the sensitivity analysis restricted to participants aged 12–17 years, both in the bivariate analysis (ρ = −0.519, *p* = 0.003) and after adjustment for perceived household financial adequacy (standardized β = −0.534, *p* < 0.001). The consistency of these findings across the primary and age-restricted analyses supports an association between greater parental monitoring and lower HbA1c in this sample. However, because of the cross-sectional design, these findings do not establish that greater parental monitoring causes improved glycemic control. Our findings are consistent with recent evidence from Türkiye. Aydın et al. reported better glycemic control with greater parental monitoring among adolescents with type 1 diabetes, although differences varied according to the monitoring dimension examined [12]. The present study extends this evidence by demonstrating that the association between parental monitoring and HbA1c persisted after adjustment for perceived household financial adequacy and remained directionally consistent in the age-restricted sensitivity analysis. Age-related differences in the burden experienced by children with type 1 diabetes and their caregivers have been reported in the literature [7]. In our study, older paternal age was associated with lower diabetes-specific family impact (ρ = −0.377, *p* = 0.002), whereas child age was not significantly associated with DIFS, CBS, PMDCS, or HbA1c. The absence of an association with child age may partly reflect the relatively narrow age distribution and limited sample size of the present study. In addition, diabetes-related family burden may be influenced more strongly by factors such as disease duration, treatment demands, family resources, and individual caregiving responsibilities than by chronological age alone.

Socioeconomic circumstances are increasingly recognized as relevant to pediatric type 1 diabetes management. In a recent multicenter study from Türkiye involving 882 families, Karakuş et al. demonstrated substantial socioeconomic inequalities in access to diabetes technologies, with household income, parental education, and regional development associated with technology use [14]. In the present study, perceived household financial inadequacy was associated with higher HbA1c and greater diabetes-specific family impact, supporting the relevance of socioeconomic circumstances not only to access to diabetes care resources but also to glycemic and family-related outcomes. Caregiver burden was also higher among families reporting income below expenses; however, although perceived household financial adequacy showed a nominally significant coefficient in the CBS regression model, the overall CBS model was not statistically significant. Therefore, the multivariable finding for caregiver burden should be regarded as exploratory and interpreted cautiously. Our findings complement recent Turkish evidence showing that caregiving for a child with type 1 diabetes can impose substantial physical, psychological, social, and financial demands on parents [13]. However, our measure reflected perceived financial adequacy rather than objective household income and should therefore be interpreted accordingly.

Parental education has been identified as a potentially relevant factor in childhood diabetes management, with recent studies linking lower parental education to suboptimal diabetes management and poorer glycemic control [25,26]. In the present study, however, maternal education was not significantly associated with HbA1c and was not significantly correlated with DIFS, CBS, PMDCS, or HbA1c in the Spearman analyses. These findings do not support a clear relationship between maternal education and the primary study outcomes in this sample.

Parental occupational patterns may also influence the context in which childhood diabetes is managed, as family structure and parental employment have been associated with diabetes-related outcomes in previous research [27]. In the present study, however, maternal and paternal employment status was not significantly associated with the primary study outcomes. Therefore, no specific effect of parental employment can be inferred from our findings.

**Figure 1:**
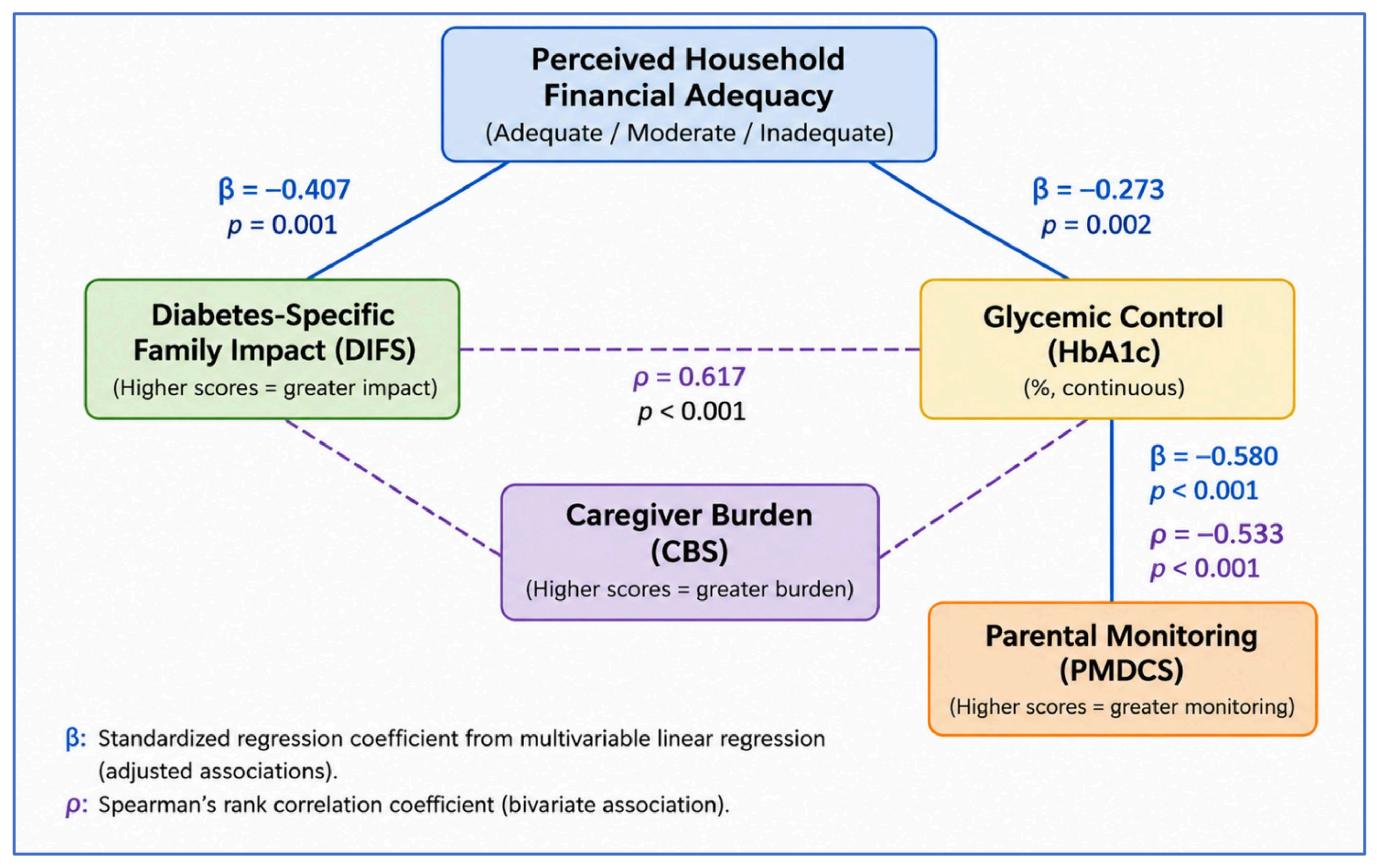
Main associations among perceived household financial adequacy, diabetes-specific family impact, caregiver burden, parental monitoring, and glycemic control. Standardized regression coefficients (β) represent adjusted associations derived from multivariable linear regression models, whereas Spearman’s ρ represents bivariate associations. Higher parental monitoring was associated with lower HbA1c in both the bivariate (ρ = −0.533, *p* < 0.001) and adjusted analyses (β = −0.580, *p* < 0.001). DIFS, Diabetes Family Impact Scale; CBS, Caregiver Burden Scale; PMDCS, Parental Monitoring of Diabetes Care Scale; HbA1c, glycated hemoglobin. Arrows represent modeled statistical associations and should not be interpreted as indicating causal directions.

Diabetes technologies may facilitate disease management and glycemic control, but their use may also introduce psychosocial challenges for children and their families [28,29]. In our sample, the use of advanced diabetes technologies was relatively limited. However, because barriers to technology access and use were not directly assessed, the reasons for this pattern cannot be determined from our data. Future studies should examine whether socioeconomic circumstances, family preferences, healthcare access, or other factors influence diabetes technology use in this population. The findings should also be interpreted within the healthcare and sociocultural context of Türkiye. Differences between countries in healthcare organization, access to diabetes technologies, socioeconomic conditions, and family caregiving roles may influence both diabetes management and the burden experienced by families. Therefore, the magnitude of the associations observed in this single-center study may not be directly transferable to pediatric diabetes populations in other healthcare settings. The main associations identified in the present study are summarized in Figure 1.

## 5. Study Limitations

This study has several limitations. First, its cross-sectional design precludes conclusions regarding the temporal or causal direction of the observed associations. Second, the study was conducted at a single tertiary referral center in northern Türkiye, which may limit the generalizability of the findings to populations with different sociodemographic characteristics, family structures, healthcare systems, and access to diabetes-related services and technologies. Third, the use of parent-reported questionnaires may have introduced reporting bias. Moreover, most questionnaires were completed by mothers, and family-level characteristics were assessed from the perspective of a single participating caregiver. No child-reported psychosocial measures were obtained; therefore, the findings may not fully capture the perspectives of the children or other family members. The Turkish version of the PMDCS was validated in parents of adolescents aged 12–18 years, whereas the present sample included children as young as 6 years. Although the association between PMDCS and HbA1c remained significant in the age-restricted sensitivity analysis of participants aged 12–17 years, the validity and measurement properties of the instrument in younger children remain uncertain. Perceived household financial adequacy was assessed using a categorical self-reported measure rather than objective household income and should therefore be interpreted as a subjective indicator of financial circumstances. The number of statistical comparisons also raises the possibility of type I error, particularly for secondary and exploratory analyses. In addition, despite the theory-driven selection of covariates, residual confounding by unmeasured clinical, socioeconomic, and family-level factors cannot be excluded. Finally, the relatively small overall sample size and some small sociodemographic subgroups may have reduced the precision and stability of subgroup comparisons and multivariable estimates. Larger, multicenter, and longitudinal studies are needed to confirm these findings.

## 6. Conclusions

This study highlights the associations of perceived household financial adequacy and family-related factors with glycemic control and family burden in children with type 1 diabetes. Perceived financial inadequacy was associated with higher HbA1c levels and greater diabetes-specific family impact and caregiver burden in bivariate and group comparisons, although the multivariable CBS model was not statistically significant. Greater parental monitoring was consistently associated with lower HbA1c in both bivariate and adjusted analyses, and this association remained significant in the age-restricted sensitivity analysis. These findings highlight the relevance of both socioeconomic circumstances and family dynamics in pediatric diabetes care. Given the cross-sectional design, the observed associations should not be interpreted as causal and should be confirmed in larger, multicenter, and longitudinal studies.

## Figures and Tables

**Table 1:** Sociodemographic characteristics of the participants.

	n (%)/Mean ± SD
Child age	10.9 ± 2.1
Gender Female Male	38 (56.7) 29 (43.3)
Mother’s age	37.5 ± 4.8
Father’s age	41.3 ± 4.9
Total number of children	2.39 ± 0.88
Mother’s education level Elementary school Middle school High school University and above	18 (26.9) 15 (22.4) 26 (38.8) 8 (11.9)
Father’s education level Elementary school Middle school High school University and above	11 (16.4) 13 (19.4) 24 (35.8) 19 (28.4)
Mother’s employment status Employed Not employed	8 (11.9) 59 (88.1)
Father’s employment status Employed Not employed	61 (91.0) 6 (9.0)
Family structure Nuclear family Large family	57 (85.1) 10 (14.9)
Perceived household financial adequacy Income below expenses Income equal to expenses Income above expenses	43 (64.2) 24 (35.8) 0 (0.0)
Place of settlement Provincial center District center Town–Village	25 (37.3) 32 (47.8) 10 (14.9)

**Table 2:** Clinical characteristics of participants.

	n (%)/Mean ± SD
Duration of DM diagnosis (years)	6.55 ± 3.01
Child HbA1c level (n = 67)	7.76 ± 1.66
Parental awareness of the child’s most recent HbA1c value Yes No	22 (32.8) 45 (67.2)
Insulin delivery modality Insulin injection Insulin pump i-Port injection port	61 (91.1) 4 (6.0) 2 (3.0)

**Table 3:** Scale scores and reliability coefficients.

Scale Scores	Mean ± SD	Cronbach α
DIFS total	18.6 ± 9.3	0.888
CBS total	31.0 ± 13.8	0.880
PMDCS total	111.0 ± 13.0	0.806

**Table 4:** Spearman correlation matrix of sociodemographic, clinical, and psychosocial variables.

Variable	1	2	3	4	5	6	7	8
1. Child age	-							
2. Diabetes duration	0.214 (0.082)	-						
3. Father age	0.130 (0.295)	−0.050 (0.685)	-					
4. Financial adequacy	−0.191 (0.121)	0.204 (0.098)	0.186 (0.132)	-				
5. DIFS	−0.063 (0.615)	−0.004 (0.972)	**−0.377 (0.002)**	**−0.330 (0.006)**	-			
6. CBS	0.065 (0.599)	−0.024 (0.845)	−0.207 (0.092)	**−0.243 (0.047)**	**0.617** **(<0.001)**	-		
7. PMDCS	−0.079 (0.527)	−0.102 (0.413)	−0.207 (0.093)	0.018 (0.887)	0.112 (0.367)	−0.144 (0.246)	-	
8. HbA1c	0.173 (0.163)	0.004 (0.972)	0.136 (0.273)	**−0.263 (0.032)**	−0.009 (0.940)	0.053 (0.672)	**−0.533** **(<0.001)**	-

Values are presented as Spearman’s ρ (*p* value). Financial adequacy was coded as 1 = income below expenses and 2 = income equal to expenses; therefore, negative correlations indicate lower values of the corresponding variable with greater perceived financial adequacy. DIFS, Diabetes Family Impact Scale; CBS, Caregiver Burden Scale; PMDCS, Parental Monitoring of Diabetes Care Scale. All analyses were based on n = 67.

**Table 5:** Comparisons between groups.

Outcome	Income < Expenses n, Mean ± SD	Income = Expenses n, Mean ± SD	Welch t	df	*p* Value	Hedges’ g	95% CI for Mean Difference
HbA1c	43, 8.14 ± 1.85	24, 7.07 ± 0.93	3.174	64.48	**0.002**	0.672	0.40 to 1.74
DIFS	43, 21.05 ± 8.55	24, 14.29 ± 9.23	2.948	44.71	**0.005**	0.759	2.14 to 11.37
CBS	43, 33.72 ± 14.35	24, 26.13 ± 11.60	2.356	56.50	**0.022**	0.559	1.14 to 14.05

Abbreviations: DIFS, Diabetes Family Impact Scale; CBS, Caregiver Burden Scale; HbA1c, glycated hemoglobin; CI, confidence interval. Welch’s independent-samples *t*-test was used because only two financial adequacy categories contained participants. Hedges’ g is reported as the effect-size estimate.

**Table 6:** Multivariable regression analyses of factors associated with HbA1c, diabetes-specific family impact, and caregiver burden.

Outcome/Predictor	B	Robust SE	Standardized β	95% CI	*p* Value
**Model 1: log(HbA1c), n = 67**					
PMDCS	−0.009	0.001	−0.580	−0.011 to −0.007	**<0.001**
Financially balanced *	−0.112	0.035	−0.273	−0.183 to −0.042	**0.002**
**Model statistics**	**Adjusted R^2^ = 0.412**	**F(2,64) = 24.12**			**<0.001**
**Model 2: DIFS, n = 67**					
PMDCS	0.073	0.071	0.102	−0.069 to 0.215	0.310
Financially balanced *	−7.848	2.341	−0.407	−12.527 to −3.169	**0.001**
Child age	−0.725	0.611	−0.166	−1.947 to 0.496	0.240
Diabetes duration	0.303	0.378	0.096	−0.453 to 1.060	0.426
**Model statistics**	**Adjusted R^2^ = 0.107**	**F(4,62) = 2.971**			**0.026**
**Model 3: CBS, n = 67**					
PMDCS	−0.078	0.121	−0.073	−0.319 to 0.164	0.522
Financially balanced *	−7.834	3.733	−0.274	−15.296 to −0.373	0.040
Child age	−0.110	1.007	−0.017	−2.123 to 1.902	0.913
Diabetes duration	0.200	0.635	0.043	−1.069 to 1.470	0.753
**Model statistics**	**Adjusted R^2^ = 0.018**	**F(4,62) = 1.296**			**0.281**

Abbreviations: B, unstandardized regression coefficient; SE, standard error; β, standardized regression coefficient; CI, confidence interval; PMDCS, Parental Monitoring of Diabetes Care Scale; DIFS, Diabetes Family Impact Scale; CBS, Caregiver Burden Scale. HC3 robust standard errors are reported. * Financially balanced indicates household income equal to expenses; income below expenses was the reference category. HbA1c was log-transformed for regression analysis.

## Data Availability

The de-identified data supporting the findings of this study are available from the corresponding author upon reasonable request, subject to applicable ethical and institutional requirements.
